# Low‐Income, Minority‐Serving Public Schools in Southern California Have Disproportionately High Surface Temperatures

**DOI:** 10.1029/2026GH001927

**Published:** 2026-09-15

**Authors:** Joaquin Murillo, Gabriella R. Dauber, Joshua B. Fisher, Gregory R. Goldsmith

**Affiliations:** ^1^ Dale E. and Sara Ann Fowler School of Engineering Chapman University Orange CA USA; ^2^ Schmid College of Science and Technology Chapman University Orange CA USA

**Keywords:** climate change, green spaces, playgrounds, remote sensing, land surface temperatures, environmental justice

## Abstract

Extensive evidence demonstrates that people from minority backgrounds often face unequal exposure to extreme heat and have less access to green space that can provide cooling relief. What is less clear is whether these inequities extend to students on school playgrounds, where high temperatures can affect physical health, social and emotional well‐being, and learning. We paired high resolution, satellite remote sensing surface temperature data for all 615 of the public schools in Orange County, California (serving ca. 450,000 students) with data on neighborhood household income, race/ethnicity of school enrollment, distance from the coast, and Normalized Difference Vegetation Index as a measure of landscape greenness. Based on >47,000 observations from the hottest late summer days, we observed mean school surface temperatures in excess of 40°C, with temperatures at some schools reaching nearly 50°C on some days. Higher school ground surface temperatures were significantly correlated with low household income (*ρ* = −0.36) and racially/ethnically diverse student populations (*ρ* = 0.37). These same schools also exhibited less variation in temperature across their grounds, which may indicate fewer spaces for students to seek relief. Greener school landscapes were weakly correlated with lower school ground surface temperatures and this was independent of socioeconomics. Our findings provide robust evidence of links between school surface temperatures and school demographics, and they highlight the growing potential of satellite remote sensing to assess heat exposure within and across schools.

## Plain Language Summary

Time spent outdoors at school, particularly during recess, is important for children's physical health, social development, and learning. However, as high temperature events are becoming more frequent due to climate change, the benefits of outdoor time for students are at risk. This study examined land surface temperatures at 615 K‐12 public schools across Orange County, California, using high‐resolution satellite data collected during the hottest days of late summer 2023. We tested whether school ground surface temperatures vary as a function of neighborhood income, race/ethnicity, distance from the coast, and landscape greenness. Results showed that schools in lower‐income communities, serving more racially and ethnically diverse populations, experienced higher outdoor surface temperatures. Schools closer to the coast and with more vegetation tended to be cooler. Our findings show that school heat exposure is unevenly distributed and may place a greater burden on students in underserved communities. This highlights the need for targeted interventions to protect children from extreme heat exposure at schools, especially in underserved communities.

## Key Points

- High resolution remote sensing reveals high variation in surface temperatures of outdoor K‐12 school spaces in Orange County, California
- Surface temperatures on the hottest days are consistent with those at which students begin to show decreased physical activity
- Schools serving lower income, more ethnically/racially diverse communities experience higher outdoor surface temperatures

## 1 Introduction

While classroom learning is a central part of the school day, time spent outdoors plays a critical role in a child's physical health, cognitive function, and well‐being. Recess provides a significant contribution to a child's daily physical activity (Erwin et al., 2012), which can positively impact health (Janssen & LeBlanc, 2010). Play also promotes social and emotional learning for children (Haapala et al., 2014; Massey et al., 2020; Pellegrini et al., 2002). In addition, time spent during recess has been demonstrated to improve cognitive ability when returning to class (Brez & Sheets, 2017), regardless of whether or not a child participated in physical activity (Castelli et al., 2014; Council on School Health, 2013).

However, the benefits of outdoor time can diminish with rising temperatures, which can limit physical activity, negatively impact learning (Park et al., 2021; Roach & Whitney, 2022), increase the risk of heat‐related illness and injury (Faurie et al., 2022; Vanos et al., 2016), and exacerbate existing health conditions such as asthma (D’Amato et al., 2015). Children are also particularly vulnerable to high temperatures compared to adults, as they possess different physiologies (e.g., take longer to acclimate to warm environments) and have less effective behavioral control and awareness over their temperatures (e.g., stopping to drink water) (Vanos, 2015). High temperate events have increased and will continue to increase with ongoing anthropogenic climate change (IPCC, 2023).

**Figure 1:**
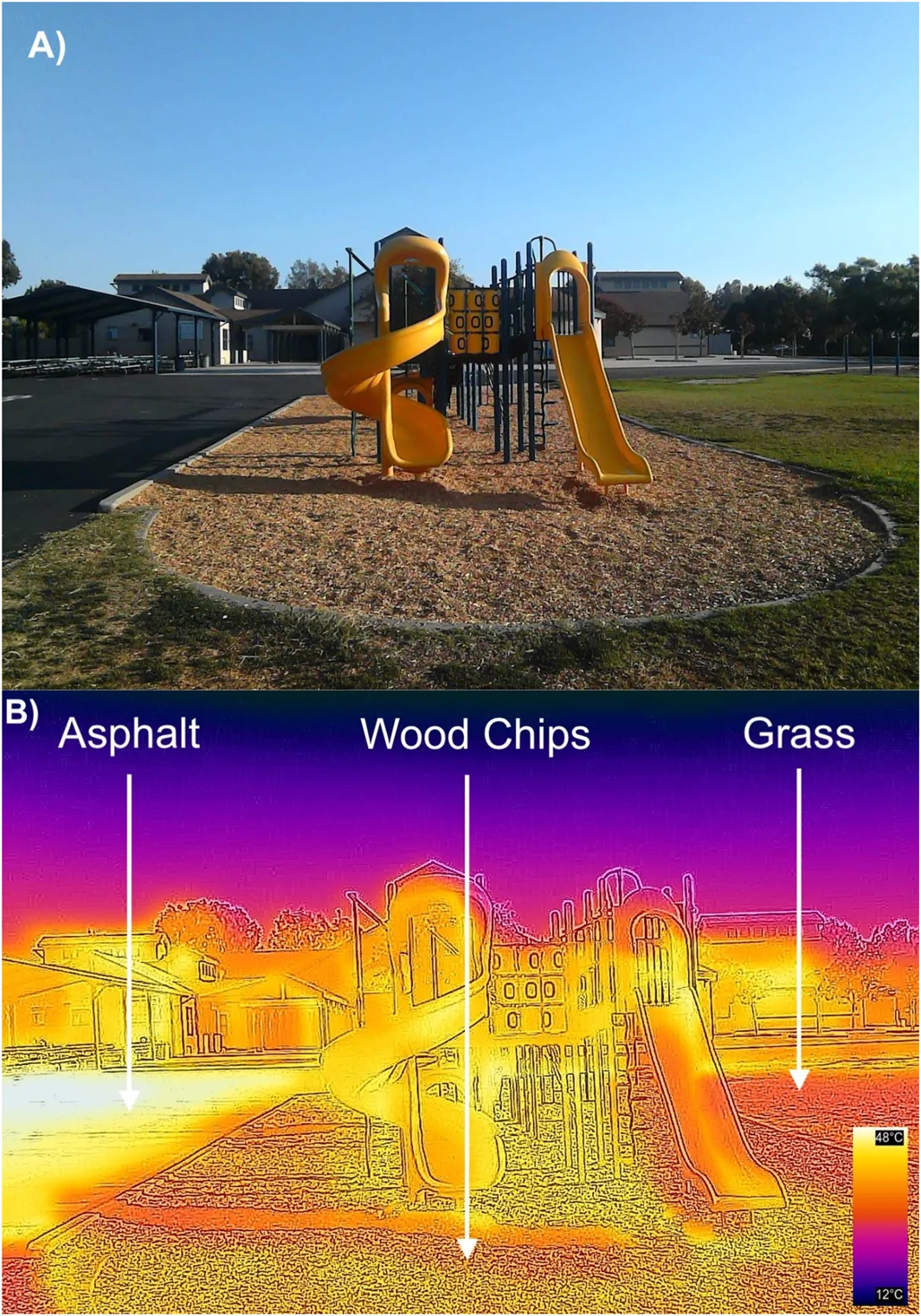
Differences in the materials used (e.g., asphalt, woodchips, grass) on school grounds affect surface temperatures. (a) True color and (b) thermal infrared photographs where lighter colors indicate higher temperatures, with peak temperatures in excess of 40°C. Images captured on 5 September 2024 at 18:00 with a FLIR ONE Pro attachment for iPhone. The atmosphere appears at colder temperatures above the surfaces in panel (b).

The design of school grounds can change exposure to high temperatures. Schoolyard layout, solar orientation, the materials used in playgrounds, and shade availability can all influence surface temperatures (Figure 1). However, not all school grounds are built equally (Agatep et al., 2026). Given evidence of disproportionate heat exposure faced by minority communities (Benz & Burney, 2021; Chakraborty et al., 2019; Fisher et al., 2026; Manware et al., 2022), schools in underserved areas may experience higher temperatures and lack infrastructure that alleviates extreme heat. Although these disparities are well documented at larger neighborhood scales, less is known about how they translate to school campuses, where children spend substantial time outdoors both during and after school hours (Young et al., 2014). Additionally, we lack studies combining data on sociodemographics and landscape composition with high resolution surface temperatures across school campuses (Jones et al., 2024).

To address this gap, we investigated how surface temperatures of school grounds vary as a function of income and race/ethnicity across all K‐12 public schools in Orange County, California (*n* = 615), one of the top 10 largest counties in the United States of America with 28 school districts serving ∼450,000 students. We focus our efforts on the hottest days of the year in order to understand how the climate extremes that are projected to become increasingly common in the years to come may drive inequities in the temperatures experienced by students of different identities. To do so, we merge satellite remote sensing land surface temperature (LST) data now available at unprecedented resolution (70 m) (Hulley et al., 2022) with common measures of income, ethnicity, geography, and green landscaping. While satellite land surface temperatures are a proxy for the air temperatures experienced by students moving around different surfaces on the landscape (Vanos et al., 2016), we expected them to be highly correlated with air temperatures and to provide insights into population‐level patterns that are challenging to obtain through intensive studies of patterns within a given school ground. Given this, we further expected schools with low‐income students and a greater proportion of racially/ethnically diverse students to not only experience higher temperatures, but also to lack outdoor areas with lower temperatures that could provide relief.

## 2 Materials and Methods

### 2.1 California School Campus Database

We identified school perimeters using shapefiles from the California School Campus Database (CSCD) (GreenInfo Network, 2021). Our data set included all K‐12 public, charter, and continuation schools in Orange County (*n* = 615 schools). Average school area was 62,690 m^2^ (median of 42,140 m^2^ with a range from 1,369 m^2^ to 318,153 m^2^).

### 2.2 ECOSTRESS Land Surface Temperature

We estimated surface temperatures for each school using data from the Ecosystem Spaceborne Thermal Radiometer Experiment on Space Station (ECOSTRESS), which provides global thermal infrared radiometer data every 3–5 days at ∼70 × 38 m resolution at nadir with an accuracy of <1 K (Hulley et al., 2022). We used the LST & Emissivity product (ECO2LSTE.001, Collection 1) to estimate temperatures for July through September 2023 and identified 5 clear‐sky daytime (between 10:00 and 18:00) scenes with the hottest median surface temperatures covering all of Orange County. Cumulatively, our sample included 47,170 temperature pixels. The average number of pixels per school was 15.3 (median of 11 pixels with a range from 1 to 80 pixels).

The CSCD provides school perimeters that encompass the entirety of the school grounds, including buildings, parking lots, playgrounds, and sports fields. To confirm that temperatures represent outdoor spaces, we masked roofs using a LiDAR‐based database of building outlines in Orange County (OC Public Works, 2021) and removed those temperature pixels from the analysis. A paired comparison of school surface temperatures with and without roofs reduced average surface temperature by only an average of 0.05°C (*t* = 1.26, *df* = 587, *p* = 0.2), indicating that the data accurately reflects outdoor surface temperatures. We performed the remaining analysis on data including roofs.

### 2.3 Air Temperature

To analyze the relationship between ECOSTRESS LST and air temperature, we retrieved hourly air temperature observations between 1 June 2019 and 1 June 2024 from three in situ, ground‐based weather stations at the (a) Irvine Tustin Ranch (grass surface surrounded by fields) from the California Irrigation Management Information System, and at the (b) Fullerton Municipal and (c) John Wayne Airports (grass surfaces entirely surrounded by pavement) from the Iowa Environmental Mesonet Database. We matched the highest quality ECOSTRESS LST observations (ECO_L2T_LSTE_002_QC_LST_accuracy_Description = <1 K (Excellent performance)) for the 70 m pixel of each weather station location to the weather station air temperature observation nearest in time.

### 2.4 Census Bureau Demographics

To analyze the relationship between school surface temperatures and community demographics, we used the B19013 (median household income), B14007 (total school enrollment), and B14007A (school enrollment for whites alone) tables developed for 2022 from the United States Census Bureau (U.S. Census Bureau, 2022). We calculated the proportion of non‐white students enrolled and refer to that throughout as racially/ethnically diverse students.

### 2.5 Distance From Coastline

To calculate the distance from each school to the coastline, we used a coastline shapefile from the NOAA Shoreline Data Explorer. We used the “Distance to Nearest Hub” Tool in QGIS v3.34, with school centroids as input points to determine distances.

### 2.6 Normalized Difference Vegetation Index

To calculate a measure of green landscaping (e.g., grass fields, tree cover) at each school, we used the Normalized Difference Vegetation Index (NDVI) from Sentinel‐2. NDVI measures the reflection of light off plant surfaces at red and near infrared wavelengths and ranges from −1 to 1 (unitless), where 1 indicates a surface with high amounts of live vegetation (Zhang et al., 2022). Sentinel‐2 provides data every 5–10 days at 20 m^2^ resolution (Main‐Knorn et al., 2017). We used the L1C B08 and B04 layers for the period overlapping the ECOSTRESS LST school temperature data, resulting in 3 days with data, from which pixels with building roofs were masked and the remaining data averaged.

### 2.7 Statistical Analysis

To analyze the relationships between ECOSTRESS LST and air temperature, we used linear regressions and calculated Root Mean Square Error and Mean Bias Error (MBE). To analyze the relationships between school temperatures and median household income, proportion of racially/ethnically diverse student enrollment, distance from coastline, and NDVI, we adopted two complementary approaches: (a) bivariate Spearman's rank correlations with *p*‐values adjusted for spatial autocorrelation using the Clifford–Richardson–Hémon method (Clifford et al., 1989) and (b) a multivariate spatial error model (Beale et al., 2010). The multivariate spatial error model was implemented using the errorsarlm function in the spatialreg package and included a spatial error term from a row‐standardized *k*‐nearest‐neighbors spatial weights matrix (Bivand et al., 2013). Prior to applying the model, we confirmed that there was low correlation among predictors by calculating variance inflation factors (VIF) using the VIF function in the car package (Fox & Weisberg, 2019). Among the predictors, only the proportion of diverse students was strongly negatively correlated to median household income (*p* < 0.001, *ρ* = −0.69). All VIFs were <2, indicating only minimal concerns of multicollinearity. Statistical analyses were performed using R (v4.4.1) (R Core Team, 2024).

## 3 Results

**Figure 2:**
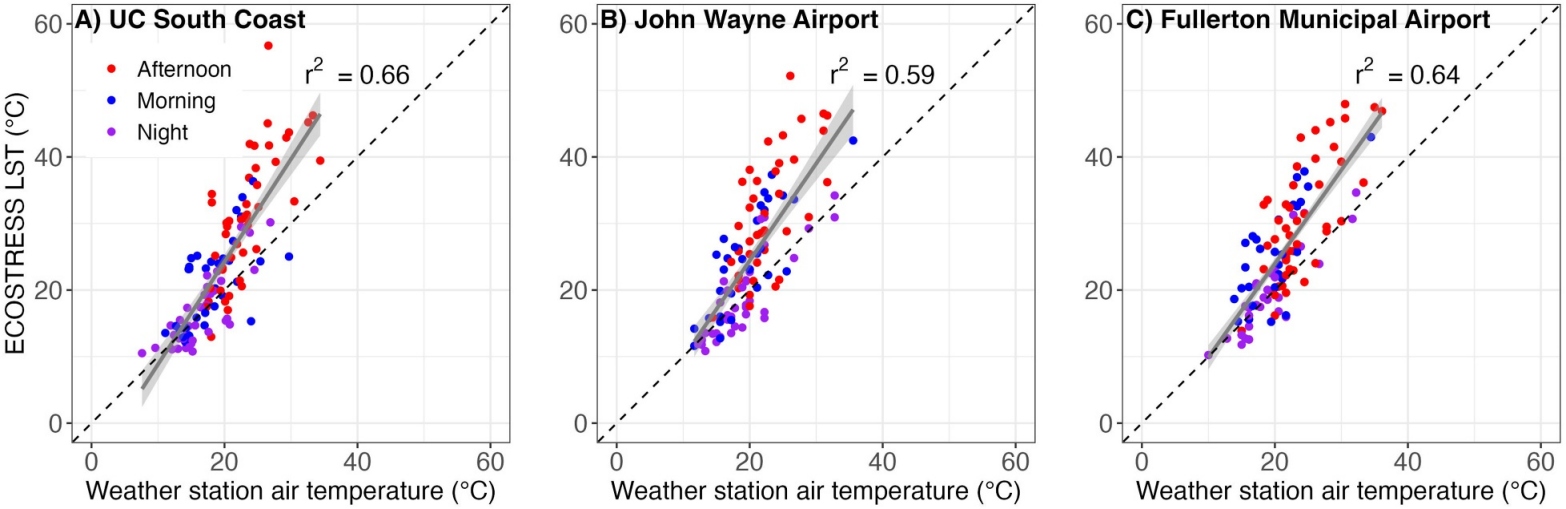
The relationship between ECOSTRESS Land Surface Temperature (LST) and air temperature at three Orange County weather stations at different times of day. LST was significantly related to air temperature (*p* < 0.001) at (a) UC South Coast Research and Extension Center (*n* = 116 observations), (b) John Wayne Airport (*n* = 110 observations), and (c) Fullerton Municipal Airport (*n* = 207 observations). The number of observations are similar among different time bins represented as different colored dots. Solid lines represent linear regressions with 95% confidence intervals and dotted lines represent 1:1.

ECOSTRESS LST significantly increased with air temperature at all three Orange County weather stations (*p* < 0.001; *r* ^2^ = 0.59–0.66; Figures 2a–2c; Table S1 in Supporting Information S1). All regression lines demonstrated a slope >1, reflecting the tendency for land surface temperatures to gradually become higher than air temperatures as the morning progresses into the afternoon. When limited to observations between 10:00 and 18:00, consistent with the times used for the analysis of school surface temperatures using ECOSTRESS, we observed similar results with similar slopes (*p* < 0.001; *r* ^2^ = 0.50–0.59; Table S2 and Figure S1 in Supporting Information S1). However, the MBE was higher between 10:00 and 18:00 (6.81–7.81°C vs. 4.09–4.56°C), which indicates that LSTs are consistently higher than air temperatures when considering only daytime hours, an effect that is likely due to the daytime storage of heat from solar radiation in surfaces (Jim, 2016).

For the hottest days in late summer 2023, public schools across Orange County ranged in their mean highest surface temperatures between 32.6°C and 45.1°C (Tables S2 and S3 in Supporting Information S1), although observations on certain days at some schools were nearly 50°C. Within‐school temperature range (max–min) was as high as 6.8°C, although mean within‐school temperature range for all schools was only 2.3°C, indicating that few schools had any outdoor spaces with lower temperatures (not accounting for spaces providing artificial or natural shading).

**Figure 3:**
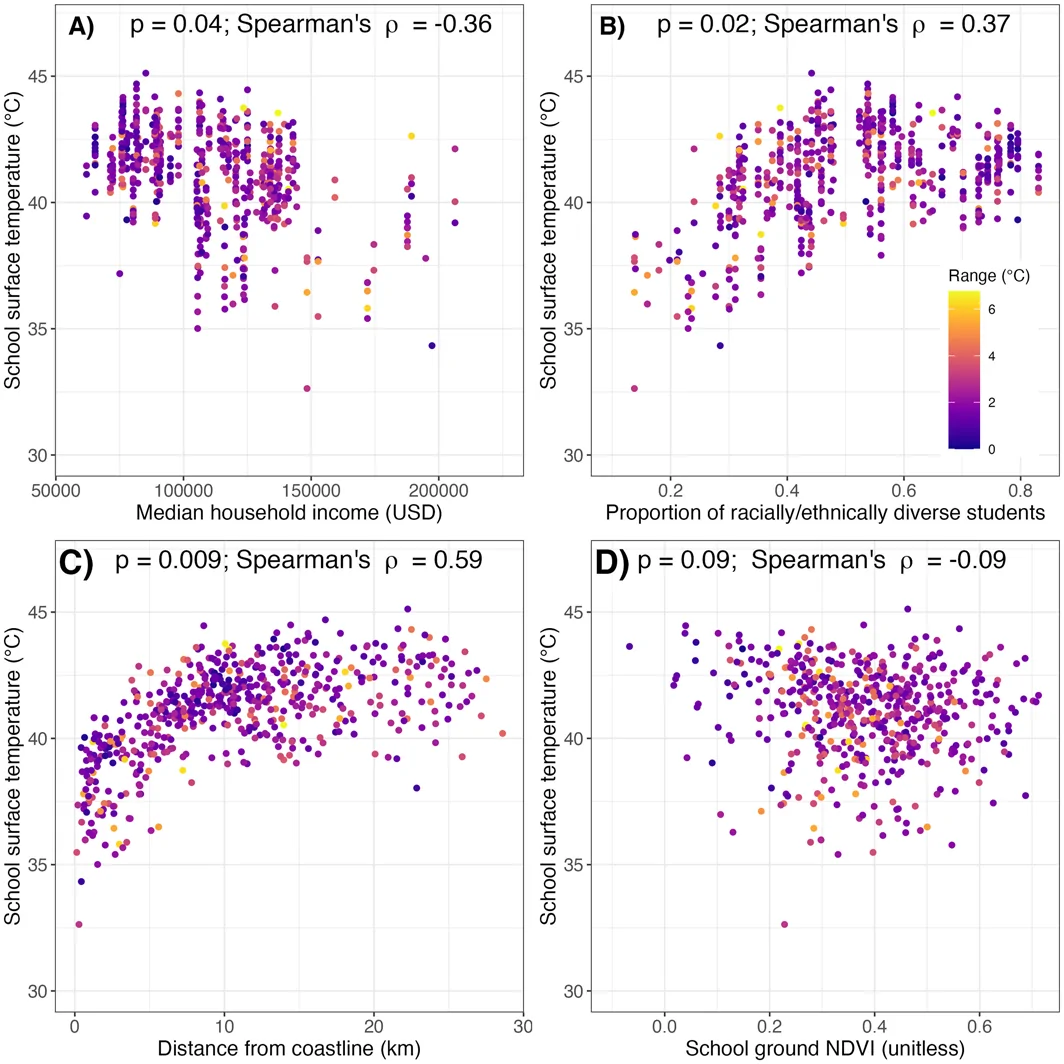
The mean surface temperatures of public schools (*n* = 615) on high temperature days in Orange County, California are significantly correlated with socioeconomic and physical factors (*n* = 5 days). Surface temperatures decreased with (a) median household income, increased with (b) proportion of racially/ethnically diverse students and (c) distance from the coastline, and did not vary with (d) Normalized Difference Vegetation Index as a measure of the green landscaping on the school grounds. Temperature ranges (max–min) observed for each school are represented by colors. A Clifford–Richardson–Hémon spatially‐adjusted *p*‐value and Spearman's rank correlation coefficient are indicated for each panel.

School surface temperatures were significantly and negatively correlated with median household income of the associated census tract (*p* = 0.04, *ρ* = −0.36; Figure 3a). Schools located in census tracts with median annual household incomes greater than the county median ($113,702) had significantly lower surface temperatures (40.4°C ± 2.0°C SD) than schools (41.5°C ± 1.6°C) in census tracts with incomes less than the county median (*t* = −7.5, *df* = 521, *p <* 0.0001). The 10 schools with the highest surface temperatures had an average median household income of $93,786 ± $16,439, well below the county median (Table S2 in Supporting Information S1). Median household income was also significantly and positively correlated with temperature range within a given school (*p* = 0.004, *ρ* = 0.25), indicating that schools in higher income neighborhoods were more likely to have a cooler location somewhere on the school grounds (Figure S2A in Supporting Information S1).

School surface temperatures were significantly and positively correlated with the proportion of racially/ethnically diverse students (*p* = 0.02, *ρ* = 0.40; Figure 3b). Schools located in census tracts with a proportion of diverse students greater than the county average (43%) had significantly higher surface temperatures (41.7 ± 1.4°C SD) than schools in census tracts with a proportion of diverse students lower than the average (39.8°C ± 2.0°C; *t* = 11.7, *df* = 338, *p <* 0.0001). The 10 schools with the lowest school surface temperatures all had populations where <30% of students were racially/ethnically diverse (Table S3 in Supporting Information S1). The proportion of racially/ethnically diverse students was significantly and negatively correlated with temperature range within a given school (*p* = 0.006, *ρ* = −0.21; Figure S2B in Supporting Information S1).

School surface temperatures were significantly and positively correlated with the distance of that school from the cooler coastline (*p* = 0.009, *ρ* = 0.59; Figure 3c). The 10 schools with the lowest surface temperatures averaged just 1.0 km from the coastline, while the 10 schools with the highest surface temperatures averaged 11 km. Notably, we found no evidence for a correlation between school distance from coastline and median household income (*p* = 0.7) and no correlation between school distance from coastline and the proportion of diverse students (*p* = 0.9).

School surface temperatures were non‐significantly and weakly negatively correlated with landscape greenness, as measured by the NDVI of the school grounds (*p* = 0.09, *ρ* = −0.09; Figure 3d). Several schools with both low NDVI and low surface temperatures were all located near the coastline. NDVI of the school grounds was not correlated with median household income (*p* = 0.1), or the proportion of diverse students (*p* = 0.8) of the associated census tracts. NDVI was not correlated with temperature range within a school (*p* = 0.4, *ρ* = −0.03).

A spatial error model indicated that the proportion of diverse students, distance from the coastline, and NDVI were all significant predictors of school surface temperatures, while the effect of median household income was nearly significant (*n* = 581, *λ* = 0.77, *z* = 24.7, *p* < 0.0001; Table 1). Standardized beta coefficients indicate that distance from the coastline (0.59) was the strongest predictor, followed by the proportion of diverse students (0.15) and both median household income (−0.09) and NDVI (−0.09). The Akaike Information Criterion of the spatial error model (1687) was lower than a standard Ordinary Least Squares model (1903), indicating that accounting for spatial autocorrelation improved the fit. A Moran's I test of the residuals from the spatial error model was not significant, indicating that the model effectively accounts for spatial autocorrelation (Monte Carlo with 9,999 permutations, Moran's *I* = −0.01, one‐sided *p* = 0.7).

Repeating the core analyses using all of the data available between 10:00 and 18:00 for July through September 2023 (*n* = 10 days) yielded very similar outcomes (Table S4 and Figure S3 in Supporting Information S1).

**Table 1:** Results of a Spatial Error Model Analyzing the Relationship Between Mean School Surface Temperatures on High Temperature Days as a Function of Median Household Income, Proportion of Diverse Students, Normalized Difference Vegetation Index (NDVI), and Distance From Coastline

Term	Estimate	Std. error	*z*	*p*‐value
(Intercept)	39.32	0.62	62.9	<0.001
Median household income	0.00	0.00	−1.7	0.085
Proportion of diverse students	1.63	0.61	2.7	0.007
NDVI	−1.10	0.30	−3.9	<0.001
Distance from coastline	0.16	0.02	7.2	<0.001

## 4 Discussion

**Figure 4:**
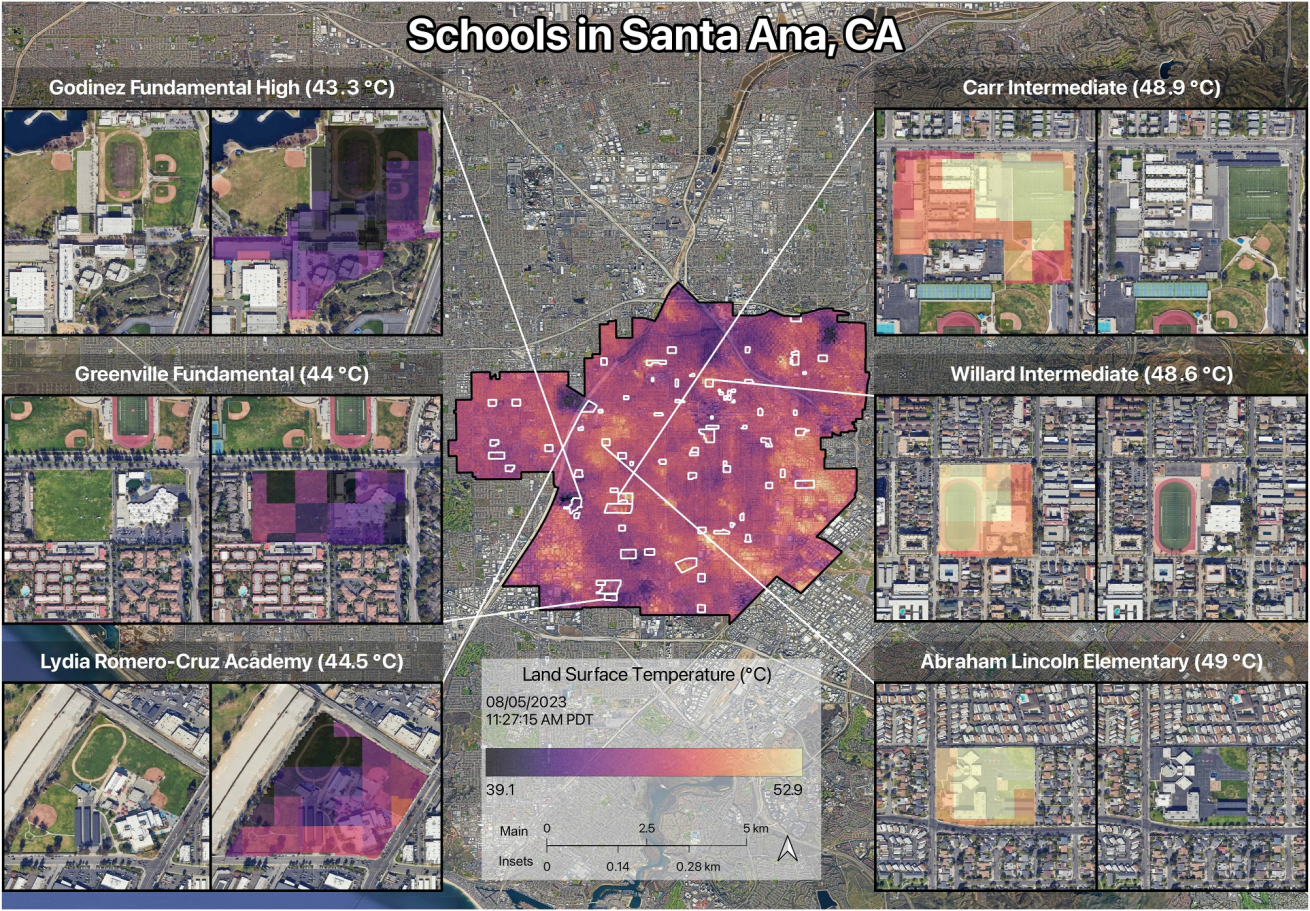
Six representative examples of cooler (left insets) and hotter (right insets) public schools from among those schools in Santa Ana, a city in Orange County, California, at midday on one of the hottest days of summer. A single school can have a considerable range of temperatures across its grounds due to variation in the surface materials (e.g., grass, synthetic turf, asphalt), shading from buildings, or the presence of trees. White boxes represent outlines of all schools in the city.

We used nearly 50,000 pixels of satellite remote sensing data from 615 schools serving nearly half of a million K‐12 students across Orange County, California to measure surface temperature patterns on high temperature days. These observations allow us to observe surface temperatures differences both within (e.g., grass playing fields vs. asphalt playgrounds) and among schools (Figure 4). Surface temperature patterns do not directly reflect the air temperatures experienced by students; however, weather station observations showed that land surface temperatures were positively related to air temperatures, indicating that ECOSTRESS captures meaningful variation in ambient thermal conditions and can serve as an important tool to complement ground‐based measurements of air temperature, wind, solar radiation exposure, and quantification of shade (Fisher et al., 2026). ECOSTRESS data cannot easily distinguish the presence of tree canopies and sail shades.

We observed that students from lower income, diverse communities were more likely to attend schools with higher outdoor surface temperatures. In particular, a 10% increase in racially/ethnically diverse students was associated with a 0.16°C increase in school surface temperature, while a $10,000 increase in income was associated with 0.05°C decrease in school surface temperatures. These schools were also more likely to have low temperature ranges, indicating less spatial variation in surface thermal conditions across school grounds. Proximity to cooler coastline temperatures was also significantly related to lower school surface temperatures, while the effects of green landscaping were slightly more equivocal, and both of these effects were largely independent of socioeconomics.

A robust body of evidence has demonstrated that lower income and minority populations experience unequal exposure to extreme temperatures (Benz & Burney, 2021; Chakraborty et al., 2019; Manware et al., 2022). In turn, temperature differences experienced by lower income, minority populations have been linked with access to green space (Agatep et al., 2026). For example, variation in surface temperature between low and high income areas has been found to strongly correlate with tree cover (McDonald et al., 2021). Our findings suggest that these neighborhood environmental inequities are also reflected at the scale of the school.

A recent analysis of the relationship between race/ethnicity and surface temperatures at schools across the United States, carried out by averaging nine 1 km pixels around each school (9 km^2^ resolution), estimated that for each 10% increase in Black and Hispanic students in a neighborhood, there was a 0.25°C and 0.38°C increase in school temperature (Jones et al., 2024). Our analysis, carried out using 70 m pixels constrained to the school perimeters (and even excluding roofs), indicates that schools with lower income and more racially/ethnically diverse populations experience hotter outdoor surface conditions. In addition, these schools exhibited lower surface temperature variability, suggesting fewer relatively cooler areas within school perimeters.

In California, there is strong evidence that property wealth and income correlate with district spending on school infrastructure (Brunner et al., 2023). Schools in lower‐income neighborhoods may be constructed with lower cost materials that retain more heat and/or lack the resources to install or maintain shade and vegetation. Buildings with low‐albedo surfaces absorb more solar radiation. Vegetation reduces surface temperatures by reducing the amount of sunlight reaching the surface under trees (i.e., shading), by increasing the return of heat to the atmosphere through plant water loss (i.e., transpiration), and by increasing the amount of sunlight reflected back to the atmosphere (Wong et al., 2021). At fine spatial scales, these characteristics can drive meaningful temperature variation, emphasizing the importance for campus‐level design interventions (O’Brien et al., 2020).

Campus‐level interventions can be complemented by specific material choices that minimize the direct risk of burns. The threshold standards for adults to be burned by hot surfaces differ among common materials, including stone aggregates such as asphalt (5 s exposure at 60°C), coated metals (5 s exposure at 60°C), plastics (5 s exposure at 74°C), and bark mulch, found on playgrounds (ISO, 2006). Nowhere is this more evident than the well‐documented differences in the burn thresholds of synthetic (5 s at 74°C) and natural grass turf (5 s at 93°C); moreover, synthetic turf has consistently been observed as having significantly higher surface temperatures than natural grass turf (Singh et al., 2024). The hottest temperatures we observed were around 50°C, but even with 70 m resolution, we may miss specific equipment (e.g., benches) at higher temperatures. The material and material colors used for equipment such as benches, tables and playground equipment must be considered (Olsen et al., 2019), although simply using lighter colored materials may not be sufficient if the material itself absorbs significant radiation energy (Kelly Turner et al., 2022).

All but one of our observed surface temperatures at schools on the hottest days were >33°C, a temperature at which children have been observed to decrease physical activity and seek shade (Lanza et al., 2022), emphasizing the importance of designing school grounds to help ease the burden of heat on students. This includes considerations of natural and artificial shade, surfaces, and materials used for equipment (Olsen et al., 2019). Natural shade may have a greater capacity to lower temperatures compared to artificial shade, but this is heavily dependent on factors such as canopy structure (Ouyang et al., 2024). Moreover, evidence suggests that children who play in environments with more vegetation and greenery experience greater focus, attention, and cognitive development (Arbogast et al., 2009; Bagot et al., 2015). Our results indicate that within‐school temperature ranges were nearly universally small, indicating that few schools are likely to be well prepared for high temperatures. TK‐6 public schools in California (and a number of other states) are required to provide at least 30 min of outdoor recess on school days, effectively guaranteeing that some students will be exposed to high temperatures (Heredia, 2024).

## 5 Conclusions

While studies have documented temperature inequities among neighborhoods, few have examined how these disparities translate to school campuses. Using high‐resolution ECOSTRESS LST observations of public schools from the hottest days of the year across the entire county, we show that schools serving lower‐income and more racially/ethnically diverse student populations experience higher surface temperatures along with less surface temperature variation within their school grounds. Surface temperatures are likely to be consistently higher than air temperature (6.8°C–7.8°C), but our results show that the two measures are strongly correlated. These results identify schools as critical sites of environmental inequity and highlight the need for equitable investments in shade, vegetation, and heat‐mitigating schoolyard design elements as high temperature events become more frequent and severe with climate change.

## Conflict of Interest

J.B. Fisher is an employee of the satellite remote sensing company Hydrosat Inc.

## Supporting information

Supporting Information S1

## Data Availability

ECOSTRESS land surface temperatures used in this study is openly available (https://appeears.earthdatacloud.nasa.gov; https://doi.org/10.5067/ECOSTRESS/ECO2LSTE.001) (Hook & Hulley, 2019). School perimeters, Census Bureau data, and building footprints are all publicly available (GreenInfo Network, 2021; OC Public Works, 2021; U.S. Census Bureau, 2022). The processed data and code used to produce the primary analyses are available in Goldsmith et al. (2026).

## References

[gh270204-bib-0001] Agatep, A. , Fisher, J. B. , Tacazon, K. , Rivera, A. , Zayas, R. , Archer, R. , et al. (2026). Thermal inequities in public parks and open spaces in Los Angeles determined by remote sensing. Npj Urban Sustainability, 6(1), 61. 10.1038/s42949-026-00366-5

[gh270204-bib-0002] Arbogast, K. L. , Kane, B. C. P. , Kirwan, J. L. , & Hertel, B. R. (2009). Vegetation and outdoor recess time at elementary schools: What are the connections? Journal of Environmental Psychology, 29(4), 450–456. 10.1016/j.jenvp.2009.03.002

[gh270204-bib-0003] Bagot, K. L. , Allen, F. C. L. , & Toukhsati, S. (2015). Perceived restorativeness of children’s school playground environments: Nature, playground features and play period experiences. Journal of Environmental Psychology, 41, 1–9. 10.1016/j.jenvp.2014.11.005

[gh270204-bib-0004] Beale, C. M. , Lennon, J. J. , Yearsley, J. M. , Brewer, M. J. , & Elston, D. A. (2010). Regression analysis of spatial data. Ecology Letters, 13(2), 246–264. 10.1111/j.1461-0248.2009.01422.x 20102373

[gh270204-bib-0005] Benz, S. A. , & Burney, J. A. (2021). Widespread race and class disparities in surface urban heat extremes across the United States. Earth's Future, 9(7), e2021EF002016. 10.1029/2021EF002016

[gh270204-bib-0006] Bivand, R. , Pebesma, E. J. , & Gómez‐Rubio, V. (2013). Applied spatial data analysis with R (2nd ed.). Springer.

[gh270204-bib-0007] Brez, C. , & Sheets, V. (2017). Classroom benefits of recess. Learning Environments Research, 20(3), 433–445. 10.1007/s10984-017-9237-x

[gh270204-bib-0008] Brunner, E. J. , Schwegman, D. , & Vincent, J. M. (2023). How much does public school facility funding depend on property wealth? Education Finance and Policy, 18(1), 25–51. 10.1162/edfp_a_00346

[gh270204-bib-0009] Castelli, D. M. , Centeio, E. E. , Hwang, J. , Barcelona, J. M. , Glowacki, E. M. , Calvert, H. G. , & Nicksic, H. M. (2014). VII. The history of physical activity and academic performance research: Informing the future. Monographs of the Society for Research in Child Development, 79(4), 119–148. 10.1111/mono.12133 25387418

[gh270204-bib-0010] Chakraborty, T. , Hsu, A. , Manya, D. , & Sheriff, G. (2019). Disproportionately higher exposure to urban heat in lower‐income neighborhoods: A multi‐city perspective. Environmental Research Letters, 14(10), 105003. 10.1088/1748-9326/ab3b99

[gh270204-bib-0011] Clifford, P. , Richardson, S. , & Hemon, D. (1989). Assessing the significance of the correlation between two spatial processes. Biometrics, 45(1), 123–134. 10.2307/2532039 2720048

[gh270204-bib-0012] Council on School Health , Ramstetter, C. , Devore, C. , Allison, M. , Ancona, R. , Barnett, S. , et al. (2013). The crucial role of recess in school. Pediatrics, 131(1), 183–188. 10.1542/peds.2012-2993 23277311

[gh270204-bib-0013] D’Amato, G. , Holgate, S. T. , Pawankar, R. , Ledford, D. K. , Cecchi, L. , Al‐Ahmad, M. , et al. (2015). Meteorological conditions, climate change, new emerging factors, and asthma and related allergic disorders. A statement of the World Allergy Organization. World Allergy Organization Journal, 8(1), 25. 10.1186/s40413-015-0073-0 26207160 PMC4499913

[gh270204-bib-0014] Erwin, H. , Abel, M. , Beighle, A. , Noland, M. P. , Worley, B. , & Riggs, R. (2012). The contribution of recess to children’s school‐day physical activity. Journal of Physical Activity and Health, 9(3), 442–448. 10.1123/jpah.9.3.442 21934153

[gh270204-bib-0015] Faurie, C. , Varghese, B. M. , Liu, J. , & Bi, P. (2022). Association between high temperature and heatwaves with heat‐related illnesses: A systematic review and meta‐analysis. Science of the Total Environment, 852, 158332. 10.1016/j.scitotenv.2022.158332 36041616

[gh270204-bib-0016] Fisher, J. B. , Rivera, A. , Cison, A. , Agatep, A. , Tacazon, K. , Spiegleman, S. , et al. (2026). Satellites, urban heat, and environmental justice: Community as the bridge between analysis and action. Environmental Justice, 19394071251413391. 10.1177/19394071251413391

[gh270204-bib-0017] Fox, J. , & Weisberg, S. (2019). An R companion to applied regression (third). Sage. Retrieved from https://www.john‐fox.ca/Companion/

[gh270204-bib-0018] Goldsmith, G. R. , Murillo, J. , Dauber, G. , & Fisher, J. B. (2026). Data and code from: Low‐income, minority‐serving public schools in Southern California have disproportionately high surface temperatures (Version v1.2) [Dataset]. Zenodo. 10.5281/zenodo.20922869

[gh270204-bib-0019] GreenInfo Network . (2021). California school campus database [Dataset]. Retrieved from https://www.mapcollaborator.org/mapcollab_cscd/

[gh270204-bib-0020] Haapala, H. L. , Hirvensalo, M. H. , Laine, K. , Laakso, L. , Hakonen, H. , Kankaanpää, A. , et al. (2014). Recess physical activity and school‐related social factors in finnish primary and lower secondary schools: Cross‐sectional associations. BMC Public Health, 14(1), 1114. 10.1186/1471-2458-14-1114 25348014 PMC4287531

[gh270204-bib-0021] Heredia, A. (2024). Senate Bill 291—Pupil rights: Recess. Retrieved from https://www.cde.ca.gov/fg/it/sb291letter.asp

[gh270204-bib-0022] Hook, S. , & Hulley, G. (2019). ECOSTRESS land surface temperature and emissivity daily L2 global 70 m (version V001) [Dataset]. NASA Land Processes Distributed Active Archive. 10.5067/ECOSTRESS/ECO2LSTE.001

[gh270204-bib-0023] Hulley, G. C. , Göttsche, F. M. , Rivera, G. , Hook, S. J. , Freepartner, R. J. , Martin, M. A. , et al. (2022). Validation and quality assessment of the ECOSTRESS level‐2 land surface temperature and emissivity product. IEEE Transactions on Geoscience and Remote Sensing, 60, 1–23. 10.1109/TGRS.2021.3079879

[gh270204-bib-0024] IPCC . (2023). Climate change 2023: Synthesis report (pp. 35–115). IPCC. Retrieved from https://www.ipcc.ch/report/ar6/syr/

[gh270204-bib-0025] ISO . (2006). Ergonomics of the thermal environment—Methods for the assessment of human responses to contact with surfaces—Part 1: Hot surfaces (version 1). International Standards Organization.

[gh270204-bib-0026] Janssen, I. , & LeBlanc, A. G. (2010). Systematic review of the health benefits of physical activity and fitness in school‐aged children and youth. International Journal of Behavioral Nutrition and Physical Activity, 7(1), 40. 10.1186/1479-5868-7-40 20459784 PMC2885312

[gh270204-bib-0027] Jim, C. Y. (2016). Solar–terrestrial radiant‐energy regimes and temperature anomalies of natural and artificial turfs. Applied Energy, 173, 520–534. 10.1016/j.apenergy.2016.04.072

[gh270204-bib-0028] Jones, K. K. , Vijay, V. , & Zenk, S. N. (2024). SchoolHEAT: Racial and ethnic inequity in school temperature. Journal of Urban Health, 101(6), 1166–1177. 10.1007/s11524-024-00919-y 39316310 PMC11652446

[gh270204-bib-0029] Kelly Turner, V. , Rogers, M. L. , Zhang, Y. , Middel, A. , Schneider, F. A. , Ocón, J. P. , et al. (2022). More than surface temperature: Mitigating thermal exposure in hyper‐local land system. Journal of Land Use Science, 17(1), 79–99. 10.1080/1747423X.2021.2015003

[gh270204-bib-0030] Lanza, K. , Alcazar, M. , Durand, C. P. , Salvo, D. , Villa, U. , & Kohl, H. W. (2022). Heat‐resilient schoolyards: Relations between temperature, shade, and physical activity of children during recess. Journal of Physical Activity and Health, 20(2), 134–141. 10.1123/jpah.2022-0405 36640783

[gh270204-bib-0031] Main‐Knorn, M. , Pflug, B. , Louis, J. , Debaecker, V. , Müller‐Wilm, U. , & Gascon, F. (2017). Sen2Cor for sentinel‐2. In Image and signal processing for remote sensing XXIII (Vol. 10427, pp. 37–48). SPIE. 10.1117/12.2278218

[gh270204-bib-0032] Manware, M. , Dubrow, R. , Carrión, D. , Ma, Y. , & Chen, K. (2022). Residential and race/ethnicity disparities in heat vulnerability in the United States. GeoHealth, 6(12), e2022GH000695. 10.1029/2022GH000695 PMC9744626 36518814

[gh270204-bib-0033] Massey, W. V. , Stellino, M. B. , & Geldhof, J. (2020). An observational study of recess quality and physical activity in urban primary schools. BMC Public Health, 20(1), 792. 10.1186/s12889-020-08849-5 32460860 PMC7251697

[gh270204-bib-0034] McDonald, R. I. , Biswas, T. , Sachar, C. , Housman, I. , Boucher, T. M. , Balk, D. , et al. (2021). The tree cover and temperature disparity in US urbanized areas: Quantifying the association with income across 5,723 communities. PLoS One, 16(4), e0249715. 10.1371/journal.pone.0249715 33909628 PMC8081227

[gh270204-bib-0035] O’Brien, D. T. , Gridley, B. , Trlica, A. , Wang, J. A. , & Shrivastava, A. (2020). Urban heat islets: Street segments, land surface temperatures, and medical emergencies during heat advisories. American Journal of Public Health, 110(7), 994–1001. 10.2105/AJPH.2020.305636 PMC7287541 32437273

[gh270204-bib-0036] OC Public Works . (2021). Orange County building footprints 2021 [Dataset]. Retrieved from https://data‐ocpw.opendata.arcgis.com/datasets/OCPW::orange‐county‐building‐footprints‐2021/about

[gh270204-bib-0037] Olsen, H. , Kennedy, E. , & Vanos, J. (2019). Shade provision in public playgrounds for thermal safety and sun protection: A case study across 100 play spaces in the United States. Landscape and Urban Planning, 189, 200–211. 10.1016/j.landurbplan.2019.04.003

[gh270204-bib-0038] Ouyang, W. , Ren, G. , Tan, Z. , Li, Y. , & Ren, C. (2024). Natural shading vs. artificial shading: A comparative analysis of their cooling efficacy in extreme hot weather. Urban Climate, 55, 101870. 10.1016/j.uclim.2024.101870

[gh270204-bib-0039] Park, R. J. , Behrer, A. P. , & Goodman, J. (2021). Learning is inhibited by heat exposure, both internationally and within the United States. Nature Human Behaviour, 5(1), 19–27. 10.1038/s41562-020-00959-9 33020588

[gh270204-bib-0040] Pellegrini, A. D. , Kato, K. , Blatchford, P. , & Baines, E. (2002). A short‐term longitudinal study of children’s playground games across the first year of school: Implications for social competence and adjustment to school. American Educational Research Journal, 39(4), 991–1015. 10.3102/00028312039004991

[gh270204-bib-0041] R Core Team . (2024). R: A Language and Environment for Statistical Computing (Version 4.4.0). R Foundation for Statistical Computing. Retrieved from https://www.R‐project.org/

[gh270204-bib-0042] Roach, T. , & Whitney, J. (2022). Heat and learning in elementary and middle school. Education Economics, 30(1), 29–46. 10.1080/09645292.2021.1931815

[gh270204-bib-0043] Singh, G. , Peterson, B. , Jay, O. , & Stevens, C. J. (2024). The effect of synthetic grass sports surfaces on the thermal environment: A systematic review. International Journal of Biometeorology, 68(7), 1235–1252. 10.1007/s00484-024-02679-5 38691211 PMC11272752

[gh270204-bib-0044] U.S. Census Bureau . (2022). 2018–2022 American Community Survey 5‐year estimates.

[gh270204-bib-0045] Vanos, J. K. (2015). Children’s health and vulnerability in outdoor microclimates: A comprehensive review. Environment International, 76, 1–15. 10.1016/j.envint.2014.11.016 25497108

[gh270204-bib-0046] Vanos, J. K. , Middel, A. , McKercher, G. R. , Kuras, E. R. , & Ruddell, B. L. (2016). Hot playgrounds and children’s health: A multiscale analysis of surface temperatures in Arizona, USA. Landscape and Urban Planning, 146, 29–42. 10.1016/j.landurbplan.2015.10.007

[gh270204-bib-0047] Wong, N. H. , Tan, C. L. , Kolokotsa, D. D. , & Takebayashi, H. (2021). Greenery as a mitigation and adaptation strategy to urban heat. Nature Reviews Earth & Environment, 2(3), 166–181. 10.1038/s43017-020-00129-5

[gh270204-bib-0048] Young, D. R. , Spengler, J. O. , Frost, N. , Evenson, K. R. , Vincent, J. M. , & Whitsel, L. (2014). Promoting physical activity through the shared use of school recreational spaces: A policy statement from the American Heart association. American Journal of Public Health, 104(9), 1583–1588. 10.2105/AJPH.2013.301461 24134355 PMC4151914

[gh270204-bib-0049] Zhang, J. , Khoshbakht, M. , Liu, J. , Gou, Z. , Xiong, J. , & Jiang, M. (2022). A clustering review of vegetation‐indicating parameters in urban thermal environment studies towards various factors. Journal of Thermal Biology, 110, 103340. 10.1016/j.jtherbio.2022.103340 36462876

