# Supplementary material for: Low‐Income, Minority‐Serving Public Schools in Southern California Have Disproportionately High Surface Temperatures
Source: Geohealth. 2026 Sep 15;10(9):e2026GH001927. doi: 10.1029/2026GH001927 (PMC13576633; doi:10.1029/2026GH001927)
Supplement: Supplementary file 1 [file GH2-10-e2026GH001927-s001.docx]

*AGU GeoHealth*

Supporting Information for

**Low-income, minority-serving public schools in Southern California have disproportionately high surface temperatures**

Joaquin Murillo^1^, Gabriella R. Dauber^2^, Joshua B. Fisher^2^, & Gregory R. Goldsmith^2^

^1^Dale E. and Sarah Ann Fowler School of Engineering, Chapman University, Orange, CA, USA

^2^Schmid College of Science and Technology, Chapman University, Orange, CA, USA

**Contents of this file**

Tables S1 to S4

Figure S1 to S3

**Introduction**

This supporting information includes supplementary tables and figures related to air temperatures and school surface temperature patterns in relation to income and race/ethnicity across public schools in Orange County, California. Surface temperatures were derived from ECOSTRESS land surface temperature data processed within school boundaries.

| **Weather station** | **Latitude** | **Longitude** | **Slope** | **R^2^** | **P** | **RMSE** | **MBE** |
| --- | --- | --- | --- | --- | --- | --- | --- |
| UC South Coast Research Extension Center | 33.68845 | -117.72118 | 1.54 | 0.66 | <0.001 | 5.67 | 4.09 |
| John Wayne Airport | 33.871543 | -117.985608 | 1.46 | 0.59 | <0.001 | 6.04 | 4.57 |
| Fullerton Municipal Airport | 33.679755 | -117.867469 | 1.41 | 0.64 | <0.001 | 5.49 | 4.56 |

**Table S1.** The relationship between ECOSTRESS land surface temperature (LST) and air temperatures at *in situ* weather stations in the study domain across all times of day (0:00 to 23:59). Reported metrics include slope, R-squared, and p-value of each linear regression, the Root Mean Square Error (RMSE) and the Mean Bias Error (MBE). The MBE is calculated as ECOSTRESS LST – air temperature (i.e., positive values indicate ECOSTRESS is warmer).

| **Weather station** | **Slope** | **R^2^** | **P** | **RMSE** | **MBE** |
| --- | --- | --- | --- | --- | --- |
| UC South Coast Research Extension Center | 1.55 | 0.59 | <0.001 | 5.83 | 7.07 |
| John Wayne Airport | 1.22 | 0.48 | <0.001 | 6.19 | 7.81 |
| Fullerton Municipal Airport | 1.18 | 0.50 | <0.001 | 5.86 | 6.81 |

**Table S2.** The relationship between ECOSTRESS land surface temperature (LST) and *in situ* air temperature at weather stations in the study domain during the day (10:00 to 18:00). Reported metrics include slope, R-squared, and p-value of each linear regression, the Root Mean Square Error (RMSE) and the Mean Bias Error (MBE). The MBE is calculated as ECOSTRESS LST – air temperature (i.e., positive values indicate ECOSTRESS is warmer).

| **School name** | **City (zip code)** | **Education level** | **Mean surface temperature (°C)** | **Median income by zip code (USD)** | **Proportion of racially/ethnically diverse students** |
| --- | --- | --- | --- | --- | --- |
| College and Career Preparatory Academy | Santa Ana (92705) | 9-12 | 44.5 | $125,059 | 0.47 |
| Edison (Thomas) Elementary School | Anaheim (92805) | K-6 | 44.2 | $81,576 | 0.54 |
| James A. Whitaker Elementary School | Buena Park (90621) | K-5 | 44.2 | $88,735 | 0.69 |
| Laurel Elementary Magnet School of Innovation and Career Exploration | Brea (92821) | K-6 | 44.1 | $114,444 | 0.46 |
| Lincoln (Abraham) Elementary School | Anaheim (92805) | K-6 | 44.7 | $81,576 | 0.54 |
| Mann (Horace) Elementary School | Anaheim (92801) | K-6 | 44.2 | $76,264 | 0.58 |
| Orange Grove Elementary School | Anaheim (92805) | K-6 | 44.5 | $81,576 | 0.54 |
| Ruby Drive Elementary School | Placentia (92870) | K-6 | 44.3 | $106,298 | 0.45 |
| Sonora High School | La Habra (90631) | 9-12 | 44.3 | $98,009 | 0.54 |
| Topaz Elementary School | Fullerton (92831) | K-6 | 45.1 | $85,220 | 0.44 |

Table S2. The 10 public schools with the highest surface temperatures on high temperature days in Orange County, California. Listed in alphabetical order.

| **School name** | **City (zip code)** | **Education level** | **Mean surface temperature (°C)** | **Median income by zip code (USD)** | **Proportion of non-white enrollment** |
| --- | --- | --- | --- | --- | --- |
| Clarence Lobo Elementary School | San Clemente (92672) | K-5 | 35.7 | $105,534 | 0.23 |
| Concordia Elementary School | San Clemente (92672) | K-5 | 35.0 | $105,534 | 0.23 |
| Eastbluff Elementary | Newport Beach (92660) |  | 35.5 | $152,700 | 0.21 |
| El Morro Elementary School | Laguna Beach (92651) | K-5 | 32.6 | $148,381 | 0.14 |
| Huntington Seacliff Elementary | Huntington Beach (92648) | K-5 | 35.8 | $116,189 | 0.30 |
| Marblehead Elementary School | San Clemente (92672) | K-5 | 35.4 | $172,056 | 0.24 |
| Moulton Elementary | Laguna Niguel (92677) | K-5 | 35.9 | $135,822 | 0.30 |
| Newport Elementary | Newport Beach (92661) | K-6 | 34.3 | $197,344 | 0.29 |
| Richard Henry Dana Elementary School | Dana Point (92629) | K-5 | 35.9 | $119,368 | 0.16 |
| San Clemente High | San Clemente (92673) | 9-12 | 35.8 | $172,056 | 0.25 |

**Table S3.** The 10 public schools with the lowest surface temperatures on high temperature days in Orange County, California. Listed in alphabetical order.

**Table S4.** Results of a spatial error model analyzing the relationship between mean surface temperatures of public schools (n = 615) on all summer days (10:00 – 18:00) for which data were available between 1 July 2023 and 1 October 2023 in Orange County, California (*n* = 10 days) as a function of median household income, proportion of diverse students, NDVI, and distance from coastline.

| **Term** | **Estimate** | **Std. error** | **z** | **p-value** |
| --- | --- | --- | --- | --- |
| (Intercept) | 35.93 | 0.80 | 44.7 | <0.001 |
| Median household income | 0.00 | 0.00 | 0.3 | 0.7 |
| Proportion of diverse students | 2.37 | 0.69 | 3.4 | <0.001 |
| NDVI | -1.17 | 0.31 | -3.7 | <0.001 |
| Distance from coastline | 0.27 | 0.04 | 7.6 | <0.001 |

**Figure S1.** The relationship between ECOSTRESS Land Surface Temperature (LST) and air temperature at three *in situ* Orange County weather stations between 10:00 and 18:00. LST was significantly related to air temperature (p < 0.001) at A) UC South Coast Research and Extension Center (n = 67 observations), B) John Wayne Airport (n = 62 observations), and C) Fullerton Muncipal Airport (n = 126 observations). Solid lines represent linear regressions with 95% confidence intervals and dotted lines represent 1:1.

Figure S2. The range (maximum-minimum) of school surface temperatures for each school as a function of A) median household income and B) proportion of racially/ethnically diverse students. Most schools had very low temperature ranges, indicating that they may lack outdoor areas with lower temperatures that could provide relief. These areas for relief were also less likely to be found at schools with low median household incomes or a high proportion of racially/ethnically diverse students. A Clifford-Richardson-Hémon spatially-adjusted p-value and Spearman’s rank correlation coefficient are indicated for each panel.

**Figure S3.** The mean surface temperatures of public schools (n = 615) on all summer days (10:00 – 18:00) for which data were available between 1 July 2023 and 1 October 2023 in Orange County, California (*n* = 10 days) as a function of (A) median household income, (B) proportion of racially/ethnically diverse students, (C) distance from the coastline, and (D) Normalized Difference Vegetation Index (NDVI). Temperature ranges (max-min) observed for each school are represented by colors. A Clifford-Richardson-Hémon spatially-adjusted p-value and Spearman’s rank correlation coefficient are indicated for each panel.
